# Molecular characterization of *Wdr13* knockout female mice uteri: a model for human endometrial hyperplasia

**DOI:** 10.1038/s41598-020-70773-w

**Published:** 2020-09-03

**Authors:** Shalu Singh, Sivapriya Pavuluri, B. Jyothi Lakshmi, Bhim B. Biswa, Bharathi Venkatachalam, Chaturvedula Tripura, Satish Kumar

**Affiliations:** 1 Centre for Cellular and Molecular Biology, Habsiguda, Hyderabad, Telangana 500007 India; 2 Department of Biotechnology, School of Interdisciplinary and Applied Sciences, Central University of Haryana, Mahendergarh, Haryana 123031 India

**Keywords:** Clinical genetics, Gene expression, Gene regulation, Genotype, Medical genetics

## Abstract

Endometrial hyperplasia (EH) is a condition where uterine endometrial glands show excessive proliferation of epithelial cells that may subsequently progress into endometrial cancer (EC). Modern lifestyle disorders such as obesity, hormonal changes and hyperinsulinemia are known risk factors for EH. A mouse strain that mimics most of these risk factors would be an ideal model to study the stage-wise progression of EH disease and develop suitable treatment strategies. *Wdr13*, an X-linked gene, is evolutionarily conserved and expressed in several tissues including uteri. In the present study, *Wdr13* knockout female mice developed benign proliferative epithelium that progressed into EH at around one year of age accompanied by an increase in body weight and elevated estradiol levels. Molecular characterization studies revealed increase in ERα, PI3K and a decrease in PAX2 and ERβ proteins in *Wdr13* mutant mice uteri. Further, a decrease in the mRNA levels of cell cycle inhibitors, namely; p21 and cyclin G2 was seen. Leukocyte infiltration was observed in the uterine tissue of knockout mice at around 12 months of age. These physiological, molecular and pathological patterns were similar to those routinely seen in human EH disease and demonstrated the importance of WDR13 in mice uterine tissue. Thus, the genetic loss of *Wdr13* in these mice led to mimicking of the human EH associated metabolic disorders making *Wdr13* knockout female mice a potential animal model to study human endometrial hyperplasia.

## Introduction

Endometrial hyperplasia (EH) is a precancerous condition for endometrial carcinoma (EC) marked with abnormal proliferation of endometrial cells^1,2^. EC is one of the most common reproductive cancers in females^3,4^ accounting for 90% of uterine cancers^5^. Although EH is a common gynecologic disease, information on the progression of EH to EC and its pathogenesis is scanty. It is now increasingly understood that modern lifestyle metabolic syndromes involving obesity, elevated estradiol, hyperinsulinemia are known risk factors for EH which generally advances to EC^4,6^. Hence, it is critical to understand the underlying mechanisms and the pathophysiology of EH. Mouse models are important tools in identifying the mechanisms underlying the development of EH as well as to test the drugs for EH treatment^7^. Some of the genetically modified mouse models available to study EH include *Beta-catenin* and *Transforming growth factor-β* knockout mice^2,5^. Genetic mouse models implicate the importance of the specific gene in the development of EH disease. However, these mice do not replicate the human EH condition that developed due to metabolic disorders such as obesity, elevated estradiol, hyperinsulinemia etc. Thus, mice which may mimic the human risk factors for EH without forcible induction would be an appropriate model to study EH and to develop drugs for EH treatment.

WDR13 protein belongs to WD-repeat containing family of proteins with diverse functions^8,9^. *Wdr13* gene is X-linked and conserved across vertebrates with 87% homology between human and mouse cDNA^10^. *Wdr13* knockout mice generated in our laboratory were viable, fertile and mildly obese in an age dependent manner. An earlier study from our laboratory has shown that hyperinsulinemia and hypertrophy of the adipose tissue were observed in the *Wdr13* knockout male mice at 12 months of age. Other than the above mentioned features, the *Wdr13* knockout male mice did not show any overt phenotype when examined thoroughly^11,12^. *Wdr13* knockout mice under stress with CCl_4_ showed increased lipogenesis and fatty liver condition. These mice also displayed hepatic hypertriglyceridemia through upregulation of PPAR pathway^13^. The phenotypic features like hyperinsulinemia, increased body and abdominal fat pad weights are known risk factors for EC^3^. In the present study, using the *Wdr13* knockout mouse model, we show the association of augmented uterine epithelial cell proliferation and the development of uterine EH. We also examined the possible involvement of estrogen in the EH development in *Wdr13* knockout mice. Further, physiological and molecular effects of the loss of *Wdr13* gene were also studied. The current findings will aid in understanding both the potential function of *Wdr13* in EH disease and also in the uterine epithelial cell division.

## Results

### WDR13 isoforms, 53 kDa and 40 kDa, were expressed in mice uteri and lack of *Wdr13* showed enlarged uteri with increased adipose and body weights

**Figure 1:**
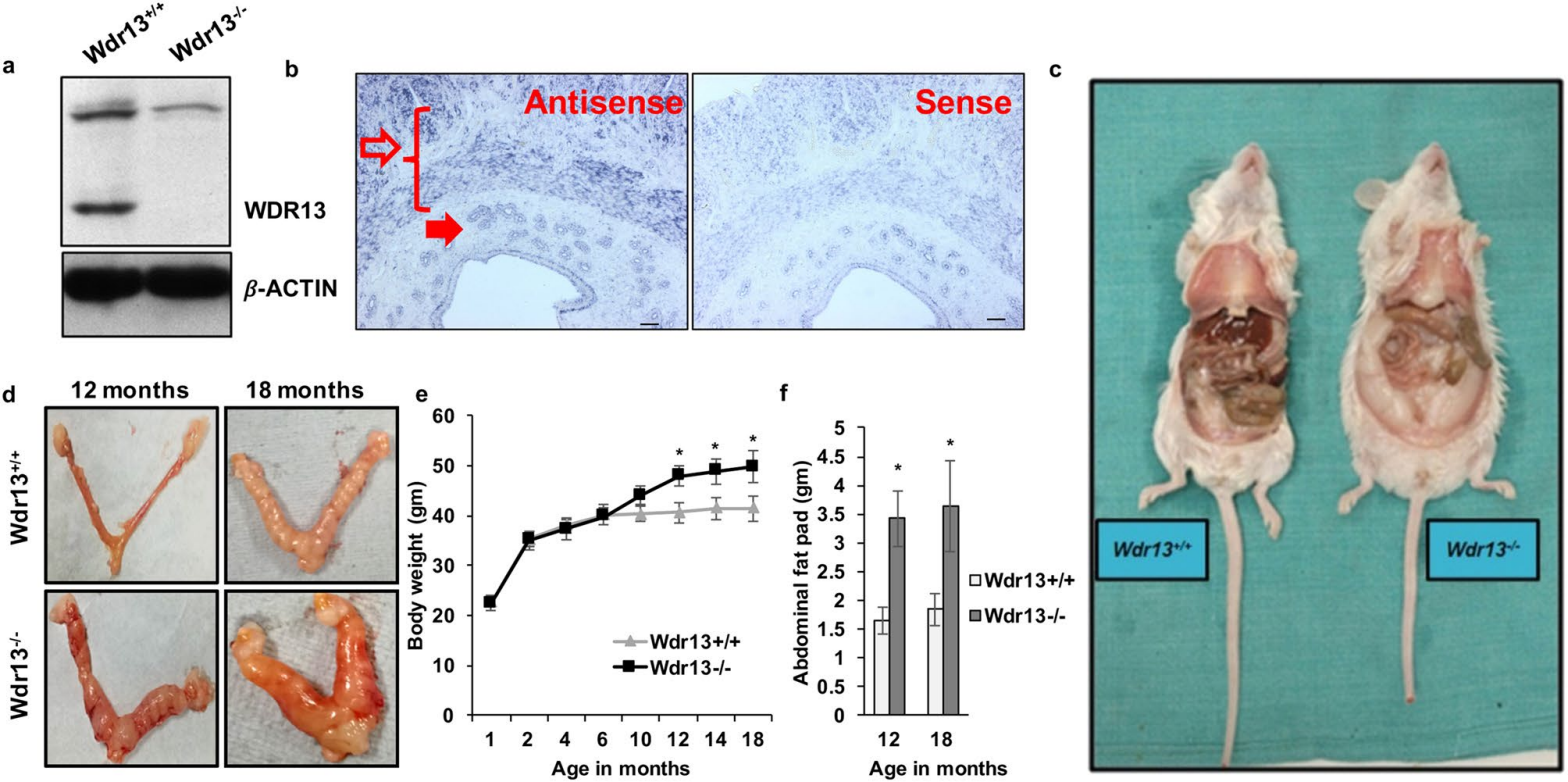
WDR13 expression in uteri and age dependent change in body and abdominal fat pad weight in *Wdr13* knockout female mice. (**a**) Two isoforms of WDR13 i.e. 53 kDa and 40 kDa are expressed in mice uteri. Earlier study^14^ has shown that the bands obtained by anti WDR13 antibody in mutant tissues is non-specific which is indicated by asterisk (*). (**b**) *Wdr13* mRNA is expressed in mouse uterine epithelial glandular cells (filled arrows) and myometrium (empty arrows) as indicated by antisense probe. The Scale bar represents 50 μm. (**c**) *Wdr13* wildtype and knockout mice picture showing mild obesity in the knockout. (**d**) Representative image of uteri of wildtype and mutant mice at 12 and 18 months of age. Knockout mice showed enlargement at both age groups. (**e**) *Wdr13* knockout female mice had higher body weight as compared to wild type mice (n = 12). (**f**) The mutant mice had significant increase in abdominal fat pad weight at 12 and 18 months of age as compared to wild type mice (n = 10).

Immunoblot analysis showed presence of 53 kDa and 40 kDa WDR13 isoforms in the uterine protein lysates of wildtype mice (Fig. 1a). WDR13 protein isoforms were not present in the *Wdr13* knockout uterine tissue but the band showed on the immunoblot near 53 kDa region was a non-specific band (Fig. 1a) as reported earlier^14^. To understand the cell specific expression of *Wdr13*, RNA in situ hybridization was performed on the murine uterine sections. *Wdr13* mRNA levels were expressed in the epithelial glandular cells and myometrium of the uterine tissue as indicated by staining with antisense probe (Fig. 1b). *Wdr13* knockout female mice showed an increased body weight (Fig. 1c) and enlarged uteri at 12 and 18 months of age (Fig. 1d). *Wdr13* knockout female mice from 12 to 18 months showed a significant increase in the bodyweight and abdominal fat pad as compared to the wild type (Fig. 1e,f). The *Wdr13* knockout mice ovulation and the estrus cycle is normal (Supplementary Fig. 1a–d) and no significant difference was found in the uterine weights between *Wdr13* wildtype and knockout mice (Supplementary Fig. 1e). In addition, *Wdr13* knockout mice also showed an increase in random insulin levels and a decrease in random glucose levels in the blood (Supplementary Fig. 2a,b). However, no significant change was observed in the levels of fasting insulin and glucose levels (Supplementary Fig. 2a,b). Further, glucose tolerance test results showed a better clearance of glucose in the knockout than in the wildtype mice (Supplementary Fig. 2c,d) indicating hyperinsulinemia condition in female *Wdr13* knockout mice.

**Figure 2:**
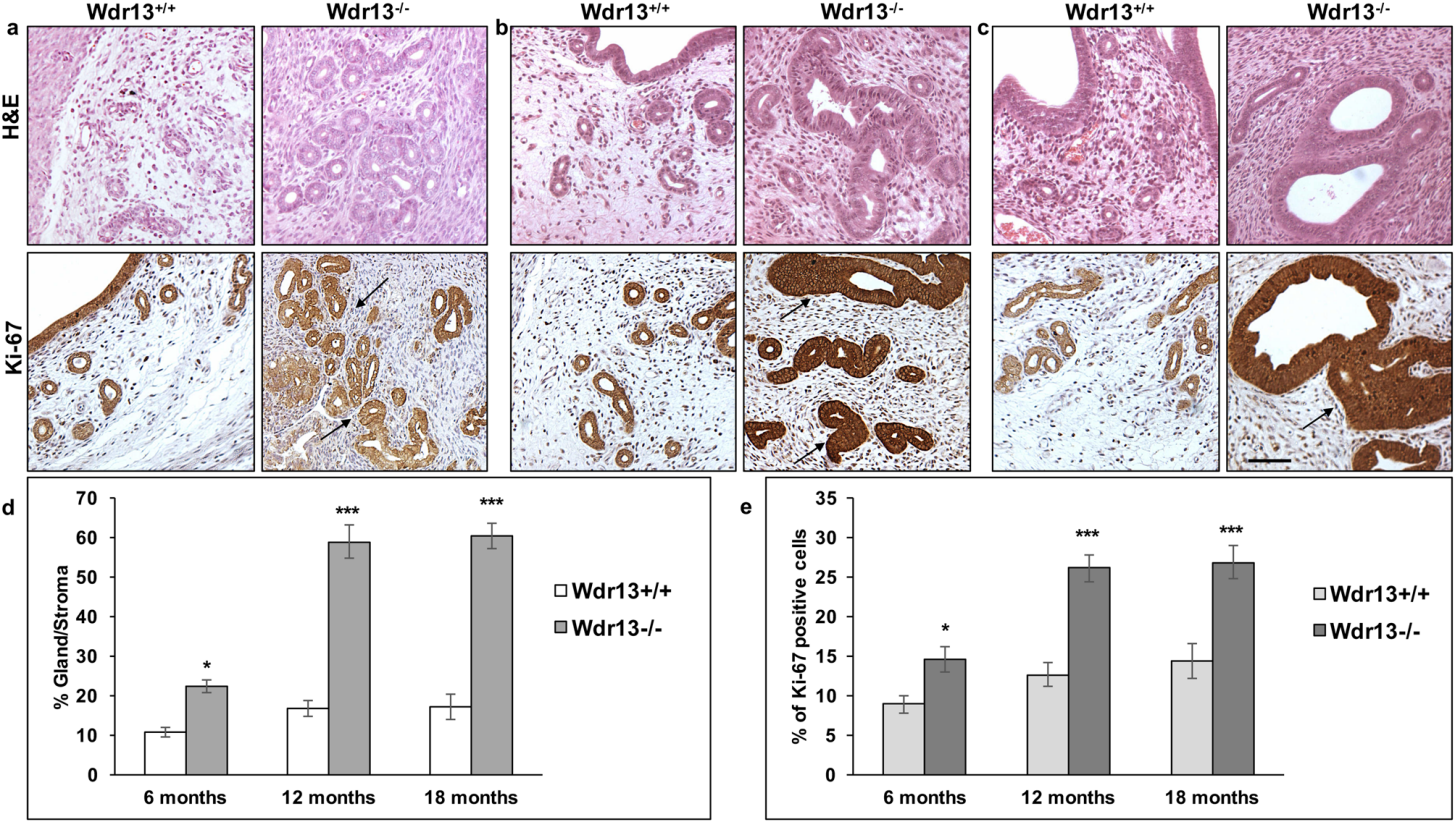
*Wdr13*^*−/−*^ mice develop endometrial hyperplasia. (**a**) Haematoxylin and eosin staining of 6 months of age mice uterine sections shows benign proliferative epithelium in glandular regions of mutant mice in comparison to those of wild type mice (n = 5). (**b**,**c**) Mutant female mice develop abnormal uterine tissue at 12 and 18 months of age. *Wdr13*^*−/−*^ uterine glands are surrounded by multilayered epithelial cells at both 12 and 18 months of age as indicated by H&E staining. Immunohistochemical analysis of Ki-67 staining showed significantly more number of positive brown nuclei (black arrows) in *Wdr13* knockout female mice uteri as compared to that of wild type mice at both 12 and 18 months of age (n = 5). (**d**,**e**) Gland to stroma ratios and Ki-67 positive cells were significantly high in mutant mice at 6, 12 and 18 months of age. Sections were observed at × 200 magnification and the scale bar represents 25 μm. The values indicated by asterisk (*) and (***) differ significantly at p < 0.05 and p < 0.001.

### *Wdr13* knockout mice showed stage-wise development of endometrial hyperplasia

*Wdr13* knockout female mice showed uteri enlargement at 12 and 18 months of age (Fig. 1d). To understand the morphological changes in the uterus, H&E staining was performed on the uterine sections at 3, 6, 12 and 18 months. The histological evaluation of the mice uteri revealed no change in the morphology at 3 months of age (Supplementary Fig. 3). However, at 6 months of age, mutant mice showed benign proliferative epithelium where an increase in the glandular epithelial cells were noticed (Fig. 2a). Although there is an increase in the number of epithelial cells in comparison to those of wild type, the hyperplastic effect was not observed (Fig. 2a). The 12 and 18 month old uteri showed hyperplastic effect with irregular distribution of the cells. Glandular epithelial cells showed shape variability with irregular nuclei and most of the cells exhibited atypical nuclear structure which is characterized by the loss of polarity (Fig. 2b,c). Gland to stroma ratio and percent of Ki-67 (cell proliferation marker) positive cells have significantly increased in *Wdr13*^−/−^ at 6, 12 and 18 months (Fig. 2d,e) indicating a stage-wise progression of EH condition. In addition, morphological examination of ovaries did not show any abnormality in the mutant mice (Supplementary Fig. 4e).

**Figure 3:**
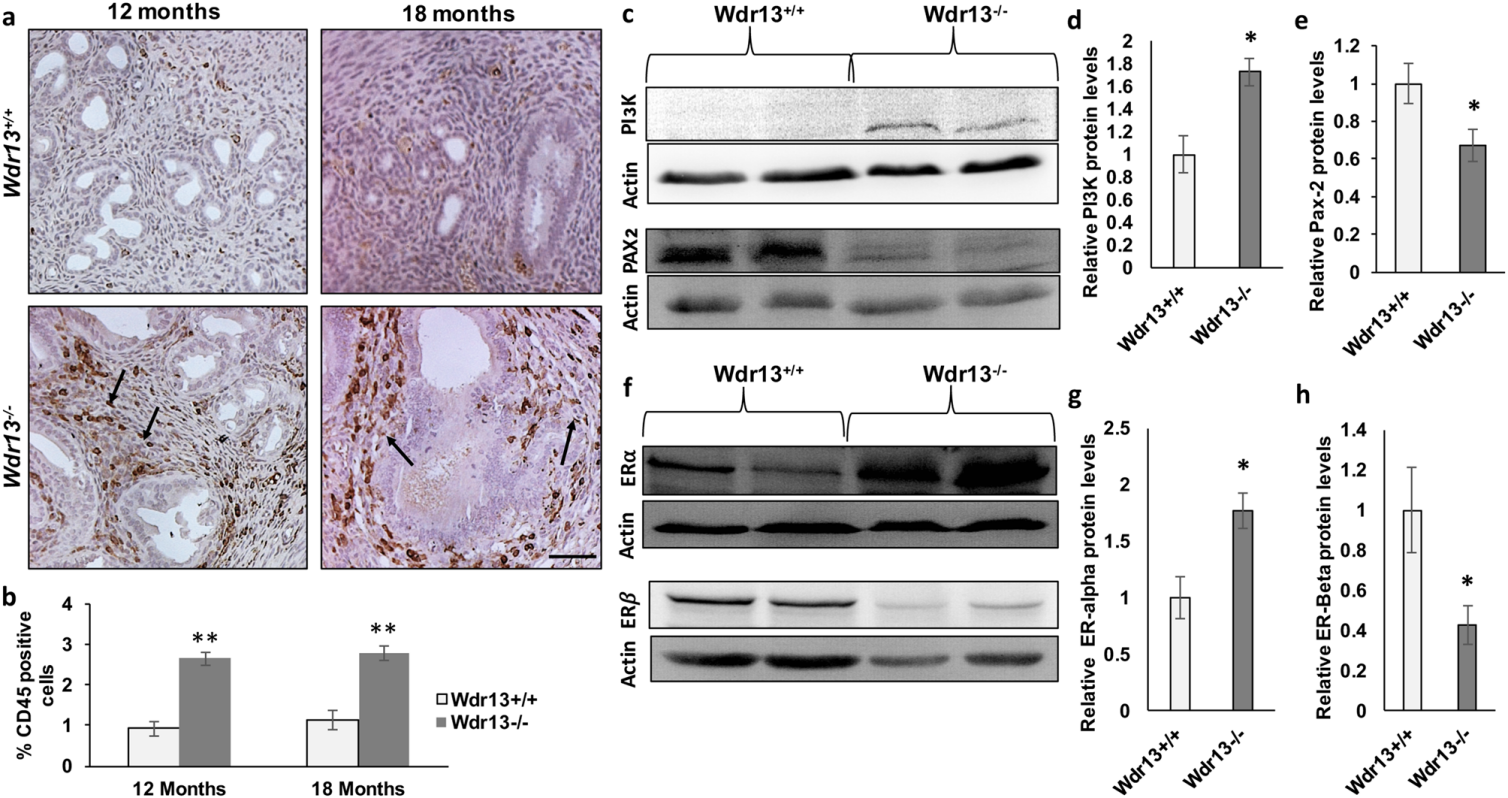
Molecular characterization studies of *Wdr13*^*−/−*^ mice uteri as a model to study endometrial hyperplasia. (**a**) CD45^+^ cells were less scattered over the stroma in mutant mice uteri. In the *Wdr13*^*−/−*^ mice, CD45^+^ cells significantly increased in number surrounding the epithelial glandular cells (black arrows). Sections were observed at × 100 magnification and the scale bar represents 25 μm. (**b**) Percentage of CD45^+^ cells calculated by Image J analysis showed significant fold increase in the *Wdr13*^*−/−*^ mice at the age of 12 and 18 months (n = 6). (**c**,**f**) Western blotting images of proteins ERα, PI3K, PAX2 and ERβ isolated from wild type and knockout mice uteri (**d**,**e**,**g**,**h**) Relative expression levels of PI3K, PAX2, ER-alpha and ER-beta in comparison to the control protein (beta actin) levels (n = 4). (**c**) The values indicated by asterisk (*) differ significantly at p < 0.05 and (**) differ significantly at p < 0.01.

**Figure 4:**
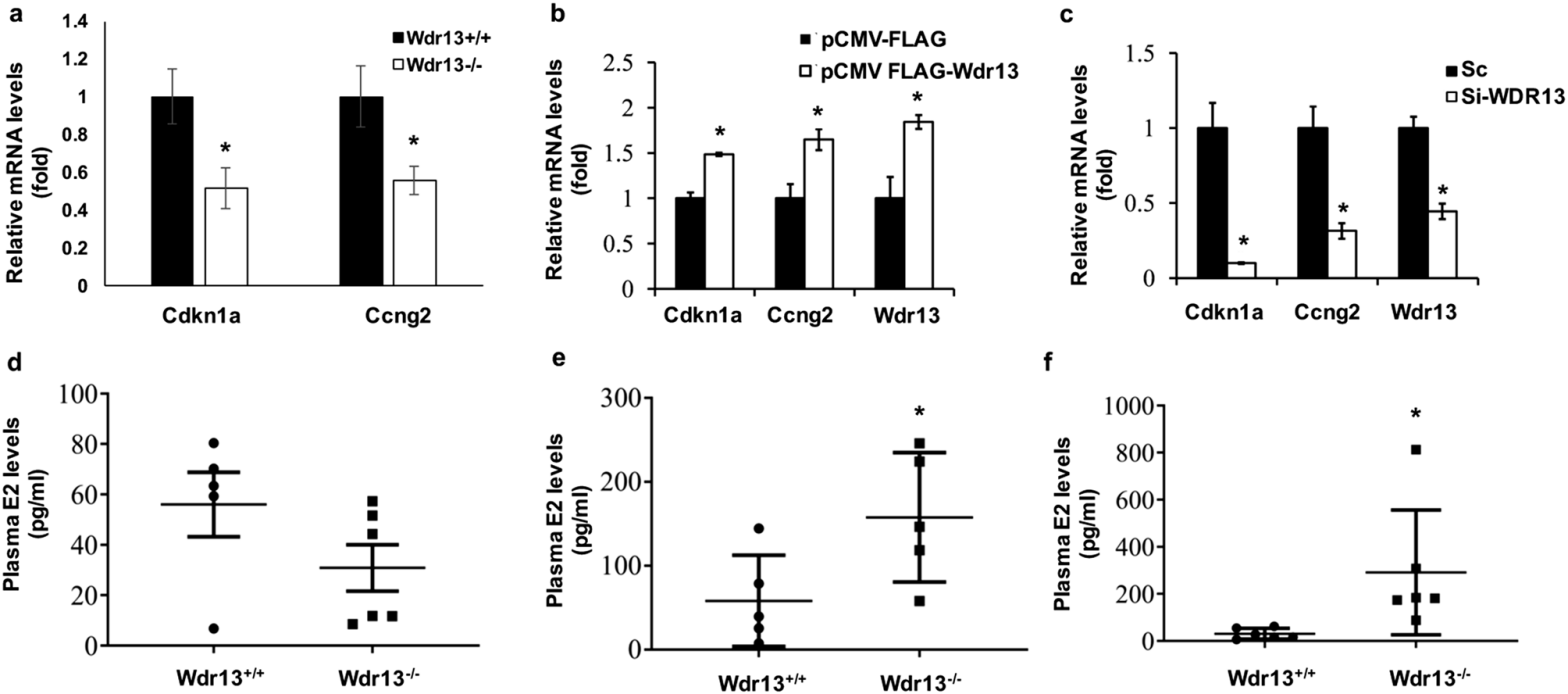
WDR13 regulates *Cdkn1a* (p21) and *Ccng2* (cyclin G2) and *Wdr13*^*−/−*^ mice showed elevated estradiol levels. (**a**) Quantitative PCR analysis showed significant downregulation of *Cdkn1a* and *Ccng2* mRNA levels in *Wdr13* mice uteri (n = 5). (**b**,**c**) Overexpression and knockdown of *Wdr13* in Ishikawa cells led to significant downregulation and upregulation in the mRNA levels of *Cdkn1a* and *Ccng2* respectively (n = 3). The values indicated by asterisk (*) differ significantly at p < 0.05. (**d**–**f**) Estradiol levels were estimated in wildtype and knockout mice at 6, 12 and 18 months of age. No significant difference in the levels of estradiol were found between knockout and wild type mice at 6 months of age. *Wdr13*^*−/−*^ mice had significantly elevated levels of plasma estradiol at 12 and 18 months of age (n = 6). The values indicated by asterisk (*) differ significantly at p < 0.05.

### *Wdr13* knockout mice uteri have increased leukocyte infiltration and have molecular abnormalities similar to human EH condition

Endometrial hyperplasia condition associates with the inflammation of uteri tissue characterized by infiltration and aggregation of CD45^+^ cells near glandular epithelium that plays an important role in increasing the formation of new epithelial cells^4,15,16^. To estimate CD45^+^ cells, immuno-histochemical analysis of uterine tissue was performed. In comparison to the *Wdr13*^+*/*+^, *Wdr13*^*−/−*^ mice showed a significant increase in the CD45^+^ cells dispersed throughout the stromal tissue (Fig. 3a,b). During proliferation, CD45^+^ cells are known to surround the glandular epithelium and this can be clearly observed in the uteri tissue of *Wdr13*^*−/−*^ mice at both 12 and 18 months of age (Fig. 3a). In addition, protein levels of ERα, PI3K, ERβ and PAX-2 which play an important role in human EH disease^17–19^ were analyzed. ERα and PI3K increased significantly in the *Wdr13*^*−/−*^ mice (Fig. 3c,f,d,g). ERβ and PAX-2 expression levels were lowered in the *Wdr13*^−/−^ mice (Fig. 3c,f,e,h). Anomaly in the expression of cell cycle regulators is often associated with hyperplastic condition^20^. Hence, mRNA levels of important cell cycle regulators (cyclins and cyclin dependent kinases) were analyzed by quantitative PCR. Cyclin G2 (*Ccng2*) and p21 (*Cdkn1a*) mRNA levels were significantly down regulated in the knockout mice uterine tissue as compared to those of the wildtype (Fig. 4a), while the other cell cycle regulators did not show any significant difference (Supplementary Fig. 5a). These results revealed that the increased hyperproliferation seen in *Wdr13* knockout uterine tissues could be due to the decreased expression of cyclin G2 and p21 which are important cell cycle inhibitors. *Wdr13* knockout mice is a whole body knockout and hence to undermine the systemic effects of the same, in vitro studies were conducted in Ishikawa cells (human endometrial adenocarcinoma cell line). To check if WDR13 regulates the gene expression of cyclin G2 and p21, overexpression and knockdown of *Wdr13* in Ishikawa cells were performed. Overexpression of *Wdr13* led to upregulation of p21 and cyclinG2 (Fig. 4b). Knockdown of *Wdr13* in Ishikawa cells led to downregulation of both cell cycle regulators (Fig. 4c). These experiments confirmed the regulatory role of WDR13 towards the expression of p21 and cyclin G2.

**Figure 5:**
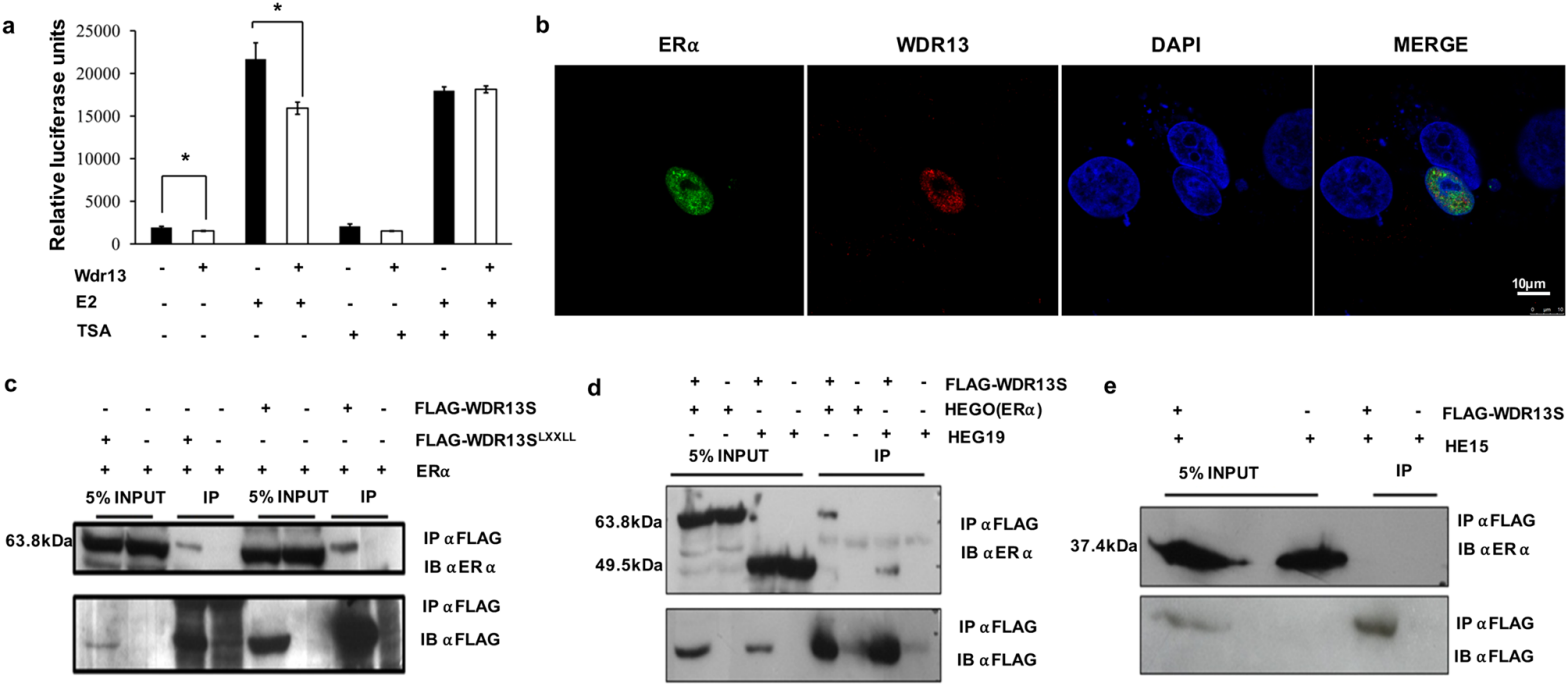
WDR13 interaction with ERα and its effect on ERα transcriptional activity. (**a**) WDR13 represses ERE reporter assay in both presence and absence of estradiol though in presence of TSA, the repression was removed. (**b**) WDR13S (Cy3, red) colocalizes with ERα (GFP, green). (**c**) Interaction of WDR13S with ERα is independent of nuclear receptor box motif wherein the anti-FLAG agarose beads pulled down ERα in both intact and mutated WDR13S overexpressing cells. (**d**) WDR13 interacts with CDEF domain of ERα (as indicated by presence of 49.5 kDa band) as well with whole ERα (as indicated by presence of 63.8 kDa band) on pull down with anti-FLAG agarose beads. (**e**) WDR13 does not interact with ABC domain of ERα (as indicated by lack of interaction with 37.4 kDa band). The values indicated by asterisk (*) differ significantly at p < 0.05. The experiments were repeated twice.

### Significant increase in plasma estradiol levels in *Wdr13* knockout mice

The observed EH in the *Wdr13* knockout female mice and the reported unopposed estrogen as a hallmark for EH^5,21^ led us to analyze plasma estradiol levels in these mice. Interestingly, the levels of plasma estradiol were significantly elevated in *Wdr13* knockout female mice as compared to that of wild type mice at both 12 and 18 months of age (Fig. 4e,f). However, there was no significant difference in the estradiol levels at 6 months of age between the wild type and mutant mice (Fig. 4d). To determine if ovaries are acting as a source of estradiol, mRNA levels of important enzymes that mediate the synthesis of estradiol were analyzed. The mRNA levels of Cyp19a1, Star (steroidogenic acute regulatory protein), Cyp17a1 (17, 20 lyase), Hsd3β (3-β-hydroxysteroid dehydrogenase), Cyp11a1 (cholesterol side chain cleavage enzyme), showed no significant difference in the expression between mutant and wild type mice ovaries (Supplementary Fig. 5b). Progesterone blocks E2 induced uterine epithelial proliferation^22^. Hence, progesterone levels were analyzed in these mice at 18 months of age and no significant difference was obtained between the levels of wildtype and mutant mice (Supplementary Fig. 5c). In addition, analysis of leptin (satiety hormone) levels also did not show any significant difference between the wildtype and mutant mice (Supplementary Fig. 5d).

### WDR13 interacts with DEF domain of ERα and acts as co-repressor for its transcriptional activity

Results from the present study showed elevated estradiol levels in the *Wdr13* knockout mice and an earlier study from our lab has shown that WDR13 interacts with ERα in HEK cells independent of the presence or absence of estradiol (personal communication, V P Singh, Satish Kumar). Estradiol is known to mediate regulation of its target genes by direct interaction with ERα^23^. Also, WDR13 is predicted to contain nuclear receptor box motif (NR box motif) as indicated by eukaryotic linear motif search (https://elm.eu.org/) as shown in Supplementary Fig. 4a. These NR box motif containing proteins acts as co-activators^24^ or co-repressors^25^ of nuclear receptors. Thus, we investigated whether WDR13 can modulate the transcriptional activity of ERα by performing estrogen response elements (ERE) luciferase reporter assay in Ishikawa cells. In this assay, overexpression of WDR13 led to the reduction of reporter activity either in the presence or absence of estradiol (Fig. 5a). WDR13 was earlier shown to interact with NCoR, HDAC1, HDAC3 and HDAC7^14^. Co-repressors often associate with HDACs in order to mediate their activity^26^. Hence, ERE luciferase reporter assay was also performed in the presence of Trichostatin A (TSA), an HDAC inhibitor. In the presence of TSA and estradiol, the ERE reporter activity gets decreased as compared to only estradiol indicating TSA inhibitory activity. However, the presence of WDR13 along with E2 and TSA does not alter the ERE activity as compared to only E2 and TSA. Thus, the repressive activity exerted by WDR13 was diminished in the presence of estradiol (Fig. 5a). These experiments suggested that WDR13 might be acting as corepressor whose activity is dependent on HDACs. Interaction of WDR13 and ERα was further confirmed by co-localization experiments (Fig. 5b). In addition, we also analyzed if the NR box motif of WDR13 was essential for its interaction with ERα by mutating the NR box motif. The co-immunoprecipitation study showed that the interaction of WDR13 and ERα was unaffected by this mutation suggesting that WDR13 interaction with ERα is independent of the NR box motif (Fig. 5c). Further, mapping of WDR13 binding on ERα was done using whole ERα (HEGO) or the independent ERα domains HEG19 (CDEF domain) or HE15 (ABC domain). It was shown that WDR13 interacted with the CDEF domain (Fig. 5d) and not with ABC domain of ERα (Fig. 5e).

### Estradiol is not the direct cause for *Wdr13* knockout uteri phenotype

**Figure 6:**
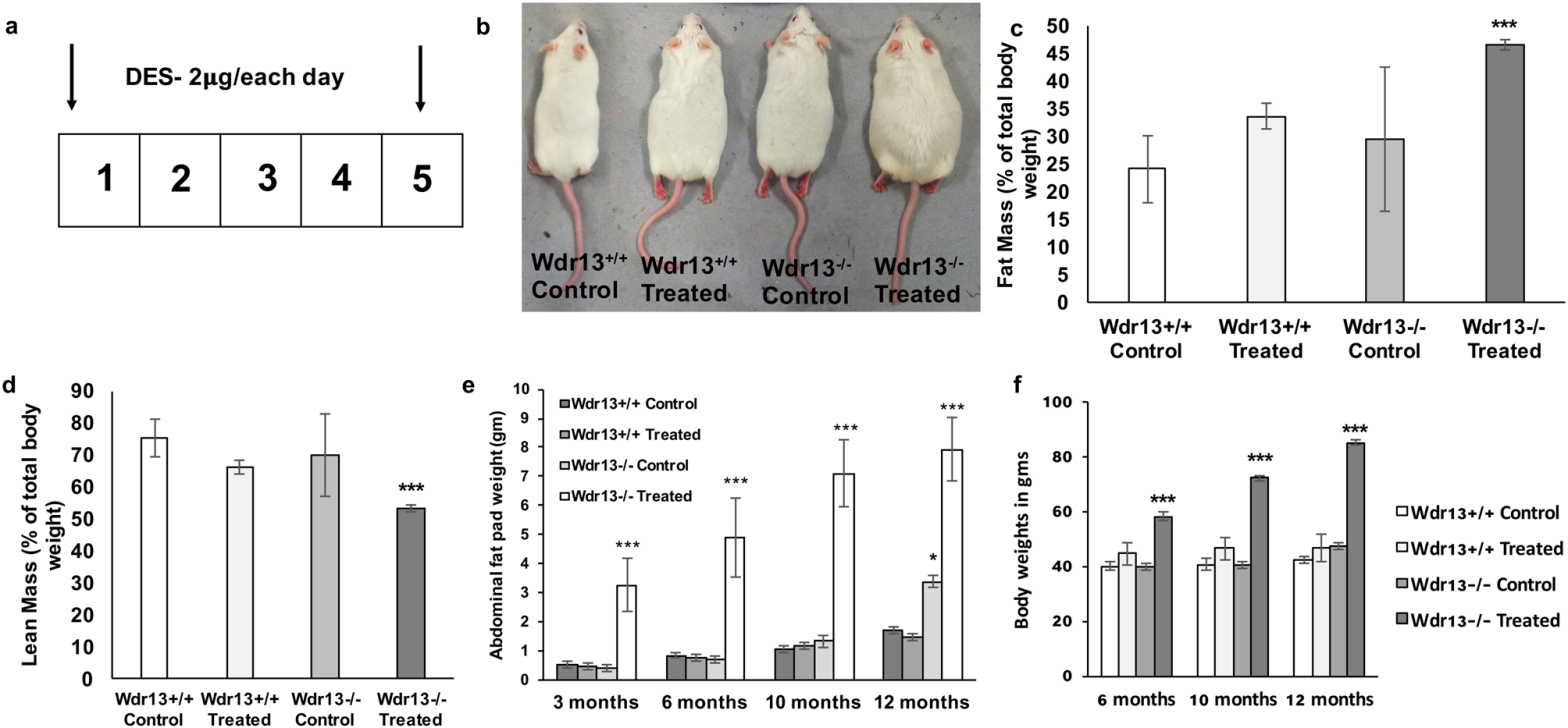
Effects of DES treatment in *Wdr13*^+*/*+^ and *Wdr13*^−/−^ mice. (**a**) Schematic representation of postnatal DES treatment (dosage of 2 μg per subcutaneous injection) from day 1 to day 5 consecutively in wildtype and mutant mice. (**b**) Representative images of *Wdr13*^+*/*+^ and *Wdr13*^−/−^ mice with and without treatment of DES where DES treated mutant mice was found obese than its counterparts at 3 months of age. (**c**,**d**) Fat mass and lean mass percentage (to total body weight) from Echo MRI analysis of *Wdr13*^+*/*+^ and *Wdr13*^−/−^ mice (n = 10) with and without treatment of DES. Mutant mice showed significant increase and decrease in fat and lean mass proportions. (**e**,**f**) Analysis of abdominal fat pad and body weights in *Wdr13*^+*/*+^ and *Wdr13*^−/−^ mice with and without treatment of DES. Mutant mice showed significant increase in abdominal fat pad and body weights. Wildtype mice treated with DES also showed increase in the abdominal fat pad weights (n = 8). Two-way Anova was performed to find the statistical significance. The values indicated by asterisk (***) differ significantly at p < 0.001 and (*) differ significantly at p < 0.05.

Unopposed E2 is a hallmark of EC^5^. Hence to undermine that the observed phenotype of mutant uteri is due to absence of *Wdr13* in uteri and not due to elevated estradiol levels, an estrogen analogue, diethylstilbestrol (DES) was injected in the wildtype and mutant mice (Fig. 6a). At 3 months of age, DES treated *Wdr13*^−/−^ mice showed significant increase in the body weight (Fig. 6b). Further, echo MRI analysis was performed to find the lean and fat mass proportions in these animals. DES treated *Wdr13*^*−/−*^ mice has significant increase in the fat mass and decrease in the lean mass proportions (Fig. 6c,d). Further monitoring of these animals indicated that significant increase in body weights and abdominal fat pad was seen in DES treated *Wdr13*^*−/−*^ mice (Fig. 6e,f).

**Figure 7:**
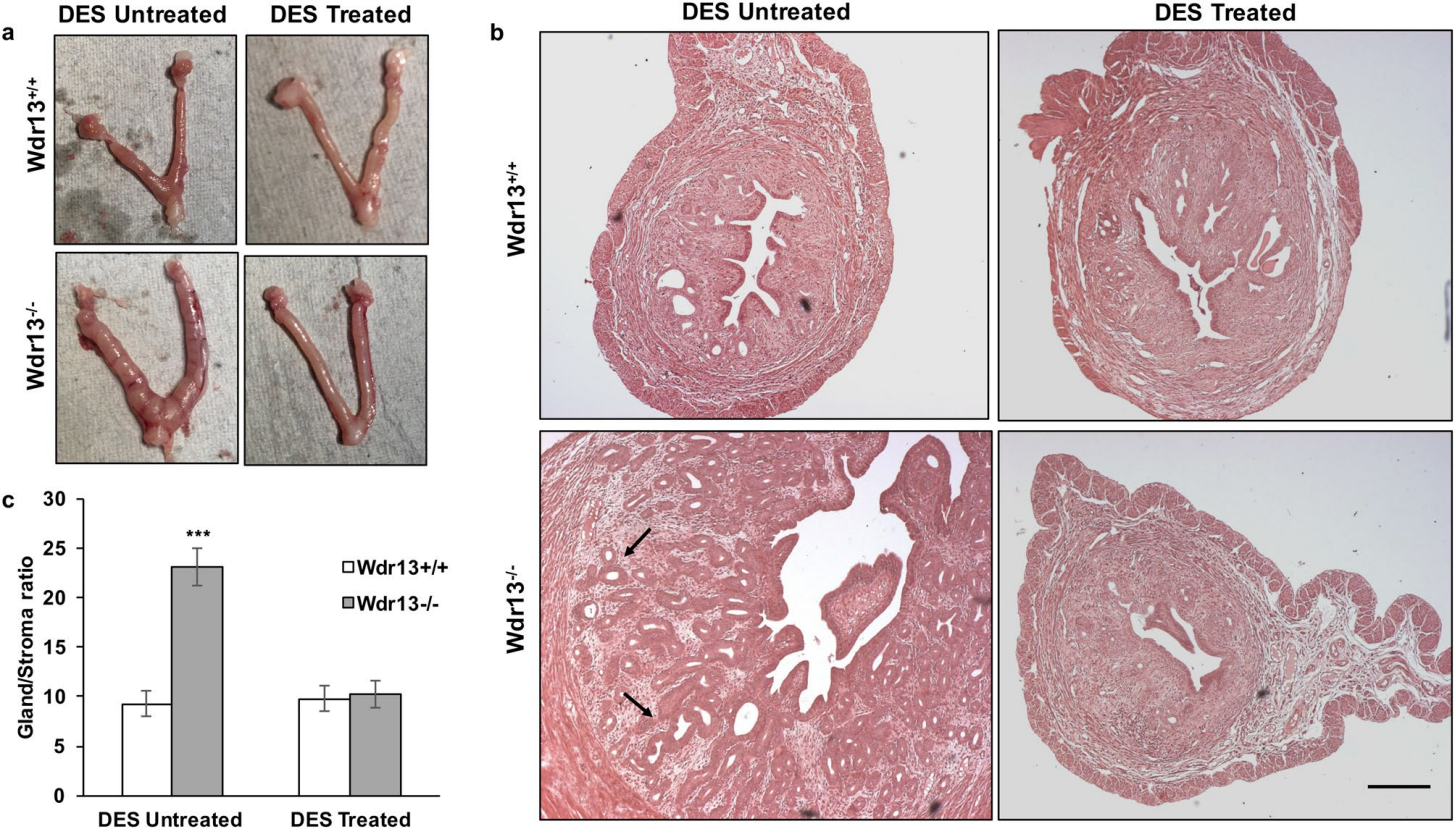
Effect of DES treatment on mutant uteri. (**a**) Uteri images of wild type and mutant mice with and without DES treatment. DES untreated mutant mice showed mild enlargement of uteri. (**b**) Haematoxylin and eosin staining of uteri sections of wild type and mutant mice with and without DES treatment. Morphology of DES treated mutant mice uteri was found to be normal. (**c**) Analysis of gland/stroma ratio calculated by Image J software where mutant mice without DES treatment showed significant increase in the gland/stroma ratio. Two-way Anova was used to find the statistical significance (n = 6). The values indicated by asterisk (***) differ significantly at p < 0.001.

However, on DES treatment, the *Wdr13* mutant uteri phenotype was found to be rescued as indicated by histo-morphological examination of the uterine tissues at 6 months of age (Fig. 7a,b) indicating the absence of estradiol’s role in the development of EH in the mutant mice. The ratio of gland/stroma was significantly more in untreated mutant mice uteri as compared to the wild type mice (Fig. 7c).

## Discussion

Endometrial hyperplasia (EH) is characterized by abnormal proliferation of the endometrial glands comprising of irregular shape and size^1^. EH is a significant problem in women and the associated risk factors include hyperinsulinemia, obesity, high estradiol levels and advanced age^5,21,27^. The mouse models that were generated to study EH lack the association of all these risk factors which are majorly lifestyle related problems in women^4,28^. There is a need for mouse model that could depict EH condition by mimicking the risk factors without forcible induction. The present study focusses on *Wdr13* knockout female mice as a potential mouse model to study EH condition that also associates with lifestyle related risk factors such as obesity, hyperinsulinemia and elevated estradiol levels.

Obesity is one of the risk factors for EH condition^21^ and in the current study, the *Wdr13* knockout female mice showed increased body and abdominal fat pad weights. Leptin resistance is one of the characteristic feature in obese mice^29^. No significant change in the levels of leptin in the wildtype and mutant mice that may be due to the mild obesity and old age of mice that involves less energy expenditure^30^. Hyperinsulinemia is as well reported to be one of the risk factor for EH and endometrial cancer (EC)^3^. In our earlier study, we found that *Wdr13* knockout male mice had increased random insulin levels at 12 months of age^11^. Similarly, a significant increase in random insulin levels was observed in *Wdr13* knockout female mice as compared to that of wild type mice (at 12 and 18 months of age). Of the several number of factors that have been reported to link hyperinsulinemia and EC, the pro-proliferative effects of insulin occupies a major role in the uteri^3^. Hence, there is a growth factor effect of increased insulin at 12 months of *Wdr13* knockout female mice on its uteri. Another important hallmark for EH is elevated estradiol^5,21^ which led us to analyze the plasma estradiol levels in these *Wdr13* knockout female mice. Apart from the age dependent obesity and hyperinsulinemia, *Wdr13* knockout female mice have elevated plasma estradiol levels. Estradiol is a known mitogen for uterine tissue and is a risk factor for EH^5,21^. Ovaries are one of the major sources of estradiol. Cyp19a1 (aromatase) is an important enzyme that plays a vital role in the synthesis of estradiol^31^. The gene expression levels of Cyp19a1 and other key E2 biosynthesizing enzymes like Star (steroidogenic acute regulatory protein), Cyp17a1 (17, 20 lyase), Hsd3β (3-β-hydroxysteroid dehydrogenase), Cyp11a1 (cholesterol side chain cleavage enzyme), showed no significant difference in the expression levels between mutant and wild type mice ovaries. In addition, ovaries showed normal morphology in both wildtype and mutant mice indicating high estradiol levels may arise due to the involvement of other local growth factors^32^. Age is also considered as one of the risk factors for the incidence of EC^33^. EC is generally aggressive in women older than 60 years and usually have higher mortality rate in comparison to the younger age group women^34^. In the present study, as the age progressed in *Wdr13* knockout mice, benign proliferative epithelium at 6 months of age transformed into EH condition in mice at 12 months of age.

EH condition, characterized by the presence of proliferative epithelial glandular cells, is one of the main risk factors for EC^2^. In the current study, mutant mice showed stage wise progression of EH condition. The benign proliferative epithelium was observed at the age of 6 months that progressed to EH at the age of 12 and 18 months with increased gland to stromal ratio. A previous study showed stage-wise progression to EH only upon the estradiol treatment given to the mice^4^. To prepone the EH condition and to study if the elevated levels of estradiol is causative factor for EH in mutant mice of the current study, diethylstilbestrol (DES) treatment was given to the wild type and mutant mice. Postnatal DES treatment leads to uterine adenocarcinoma in CD1 mice in an age dependent manner^35^. We hypothesized that postnatal treatment of DES might prepone the EH condition in mutant mice. On the contrary, the results showed a rescue of EH phenotype in DES treated mutant mice. These observations indicated that estradiol is not leading to the development of EH in these mutant mice. Mutant mice showed benign proliferative epithelium at 6 months of age where the estradiol levels were found to be normal indicating that it is not directly responsible for the EH condition. Other than estradiol, several metabolic impairments are also responsible for the cause of EH condition^36,37^. *Wdr13* mutant mice has shown impairment related to lipogenesis through PPAR pathway^13^. Future studies are required in understanding the molecular mechanisms behind rescue of the phenotype in DES treated *Wdr13*^*−/−*^ mice.

Estrogen receptor-alpha (ERα) plays an important role in the cellular proliferation of the endometrium^23^. ERα protein levels were significantly increased indicating its high transcriptional activity in mutant mice uteri. ERα regulates proliferation in its target organs such as the uteri^23^ by recruiting coactivators and corepressors^38^. Our study clearly suggests that WDR13 functions as a corepressor of ERα transcriptional activity, as it interacts with ERα and represses ERE reporter activity. It is already known that unliganded ERα recruit corepressors and HDAC complexes to keep target genes in repressed condition^26^. On the other hand, estradiol bound ERα stimulates cell proliferative genes and represses anti-proliferative genes by recruiting co-activators and co-repressors respectively^39^. Hence, there is a possibility that WDR13 might be acting as an important co-repressor in estradiol dependent and independent ERα transcriptional activity and its absence in the uterine tissue might have led to increased expression of pro-proliferative genes. However, further experiments are required to identify these target genes of WDR13. WDR13 has NR box motif, which is present in co-repressors^40^ that generally is involved in the interaction with nuclear receptor via these NR motif(s)^24^. In the present study we found that WDR13 interacts with ERα at the DEF domain of the NR motif. Using ERα specific inhibitors such as methyl-piperidino-pyrazole^41^ might give a clear understanding if ERα has a direct role in EH condition of the mutant mice. In addition, the downregulated expression of the cell cycle inhibitors, cyclin G2 and p21, implicated a regulatory role of WDR13 during cell cycle process which was supported by our knockdown and overexpression studies. Cyclin G2 and p21 genes are not only expressed at low levels during cell division^42,43^, but also found to be downregulated in cancers^44,45^. Our earlier study, showing WDR13 interaction with p21 promoter further strengthens the fact that WDR13 regulates p21^11^. Thus, the loss of WDR13 in mice uterine tissue may have led to dysregulation of these cell cycle inhibitors contributing to EH in these mice.

In the current study, we have analyzed the expression of proteins associated with the etiology of EH in human patients. Studies have indicated that increased ERα expression in hyperplasia then decreases stepwise as disease progresses^46,47^. ERα expression levels were high in mutant mice uteri. Another clinically relevant marker is PAX2 where many studies showed its loss of expression during EH condition^18^. The mutant mice uteri also showed decreased PAX-2 expression. PI3K is overexpressed in most of the human EH cases^17^ and in the present study PI3K levels were high in the knockout mice. Interestingly, the recent study showed that WDR13 inhibited PI3K/AKT pathway^48^ indicating that the lack of *Wdr13* in mutant mice might have increased the PI3K levels. ERβ, a known inhibitor of ERα activity, observed to have a decreased expression in human EH condition^19^, was also found to be downregulated in the mutant mice. The protein expression profile pattern of ERα, PI3K, ERβ and PAX2 in mutant mice uteri was similar to that found in human EH condition^17–19^ reinstating that the *Wdr13* mutant mice can act as a model for EH condition. Abnormal proliferation of the endometrium leads to increase in the CD45^+^ cells. Also, increased leukocyte infiltration is a pathological condition, which is one of the important characteristic features of EH condition in humans^4^. The present study’s findings also show consistent results, where the CD45^+^ cells infiltrate in the stroma and are dispersed around the glandular epithelial lobes. Taken together, our findings recapitulate the clinical findings seen in human patients suggesting its pivotal role in the drug studies.

In conclusion, the present study has shown for the first time the presence of WDR13 isoforms (53 and 40 kDa) in the mice uteri and its absence leads to hyperplasia of endometrial epithelial glandular cells. The current study indicated the novel function of WDR13 as a corepressor of ERα and the anti-proliferative functions of WDR13 is mainly demonstrated through its regulation of *Cdkn1a* and *Ccng2* expression. The *Wdr13* knockout female mice are obese, have increased estradiol, increased adipose fat pad weight and are hyperinsulinemic. These factors are important known risk factors for EH^3,21^ that might be contributing to EH condition observed in the *Wdr13* knockout female mice. Expression profile of important proteins like ERα, ERβ, PI3K, PAX2 and CD45^+^ found in human EH also correlated with these mutant mice indicating close resemblance of these *Wdr13*^*−/−*^ mice to human EH condition. Human protein atlas also indicates the deregulated expression of WDR13 in endometrial cancer^49^. While ECs are linked to metabolic syndromes including obesity and hyperinsulinemia^27^, unopposed estradiol has been reported as the hallmark for EC^5^. There is an ever increasing demand for mouse models, which could mimic human EH and thus could be used for studying drugs used to treat EH^7^. A mouse model which could recapitulate these metabolic syndromes along with EH would be best for studying drugs for treating EH. Taken together, uterine hyperplasia accompanied by increased estradiol, hyperinsulinemia and obesity in these *Wdr13* knockout female mice, makes them a good model to study EH condition.

## Methods

### Animal maintenance

The present study was approved by Institutional Animal Ethics Committee (Animal trial registration number 20/1999/CPCSEA dated 10/3/99) of the Centre for Cellular and Molecular Biology, Hyderabad, India. All mice experiments were performed in accordance with the approved institutional ethical guidelines of Centre for Cellular and Molecular Biology, Hyderabad, India (project numbers IAEC74/CCMB/2019, IAEC75/CCMB/2019). *Wdr13* wild type mice and *Wdr13* knockout mice were housed in 12 h light–dark cycle and were fed ad libitum^11^. Random cycling mice were utilized for the study.

### Histology, immunohistochemistry and RNA in situ hybridization

Uteri, fixed in 4% paraformaldehyde, embedded in paraffin wax were used for histological examination. Four µm thick sections were mounted on positively charged slides (Fischer scientific, catalog no. 22230900, USA). The sections were stained with haematoxylin and eosin to study tissue histology. Immunostaining was performed using Ki67 antibody (Millipore, catalog no. AB9260, rabbit polyclonal, USA), ERα antibody (Santa Cruz, catalogue no. sc543 (HC-20), USA) and CD45 (Santa Cruz, catalogue no. sc28369, USA). Immunostaining was performed as per manufacturer’s guidelines (BD Biosciences diaminobenzidine substrate, catalog no. 550880, USA). Zeiss AxioImager imaging system was used to capture images and the positive cells were counted manually using Axioskop (AxioVision software). RNA in situ hybridization was performed using DIG RNA labeling kit (Roche applied sciences, cat no 11175025910) as previously described^50^. H&E staining of 6 month uteri sections were captured at 100 × magnification and ERα immunostaining images were captured at 40 × magnification on Zeiss AxioImager imaging system. CD45^+^ immunostaining images were captured at 100 × magnification. Analysis of gland/stroma was performed as described earlier^4^. Random fields (n = 10) were selected from each mice uterine section and the area of stroma and glands were calculated for each field using ImageJ software.

### Western blot and quantitative PCR

Uteri tissue were snap-frozen and stored in − 80 °C until further use. For western blotting, uteri were lysed in RIPA lysis buffer. The lysate was quantified using BCA Protein Assay Kit (Thermo Scientific, catalog no. 23225, USA), separated on 10% SDS PAGE, and blotted on PVDF membrane. Anti WDR13 antibody (Sigma, catalog no. HPA000913, USA), anti ERα antibody (Santa Cruz, catalogue no. sc543 (HC-20), USA), anti PAX-2 antibody (Santa Cruz, catalogue no. sc-130387, USA), anti PI3K antibody, anti ERβ antibody (Sigma, catalogue no. E1276, USA and anti-beta actin antibody (Santacruz, catalog no. sc-47778, USA) were utilized for western blot analysis. A protein amount of 30 µg was loaded on SDS PAGE and transferred on to the nitrocellulose membrane. Beta actin is used as a loading control for western blotting (Full blot images were represented in the Supplementary Fig. 6 and Supplementary Fig. 7). Quantitation of protein expression was carried out using Image J software.

Trizol (Thermo Fisher, catalog no. 15596026, USA) was utilized to isolate total RNA from cells, uteri and ovaries. Reverse transcription was performed using reverse transcriptase kit (Promega, catalog no. A3800, USA). SYBR Green (Takara, catalog no. RR820A, Japan) was used to perform quantitative PCR as described earlier^14^. For uterine tissue ribosomal protein L13 (Rpl13a) was used as a reference gene^51^. For ovarian tissue ribosomal protein L19 (Rpl19) was used as a reference gene^52^ For experiments in Ishikawa cells human ribosomal protein 36B4 was used as reference gene^53^. Primers sequences used in the study are mentioned in the Supplementary Table 1.

### Cell lines maintenance, overexpression and knockdown studies

Ishikawa cells^54^ were maintained in DMEM supplemented with 10% fetal bovine serum. For experiments, Ishikawa cells were grown in phenol red free DMEM supplemented, with 5% charcoal stripped serum for two passages, and seeded in the required culture dishes. For overexpression studies, 500 ng of Flag-WDR13^14^ was transfected in 24 well plate by Lipofectamine 2000 Transfection Reagent (Thermo Fisher, catalog no. 11668027, USA) using the manufacturer’s instructions. For knockdown experiments, 100 pM of siWDR13 (Santacruz, catalog no. sc-155258, USA)/Scrambled siRNA (Santacruz, catalog no. sc-37007, USA) was transfected in 24 well plate using RNAiMAX transfection reagent (Thermo Fisher, catalog no. 13778075, USA) using the manufacturer’s instructions. Cells were lysed after 72 h of transfection in Trizol (Thermo Fisher, catalog no. 15596026) for RNA isolation, followed by cDNA isolation. Quantitative PCR was thereafter performed.

### Co-immunoprecipitation and co-localization

Co-immunoprecipitation assay was performed in HEK cells. Cells were lysed in lysis buffer (50 mM Tris HCl, 150 mM NaCl, 1 mM EDTA, 1% Triton X-100 and Protease inhibitor cocktail) and pull down was performed by using anti-FLAG agarose beads (Sigma, catalog no. F2220, USA). Immunoblotting was performed with anti ERα antibody (Santa Cruz, sc-7207 or sc-543, USA) or anti-FLAG antibody (Sigma, catalog no. F3165, USA). HEGO encoding 63.8 kDa whole ERα with five domains i.e. A/B, C, D, E, F; HEG19 (CDEF domain of ERα) encoding 49.5 kDa protein and HE15 (A/B, C domain of ERα) encoding 37.4 kDa protein were used^55^. FLAG-Wdr13 vector encoding 53 kDa WDR13 isoform, and FLAG-Wdr13S encoding 43 kDa WDR13 isoform^14^ were used. Mutation of the LXXLL motif of the 43 kDa WDR13 isoform (FLAG-Wdr13S) to LXXAA motif (FLAG-Wdr13SLXXLL) was achieved by site directed mutagenesis (SDM) using phusion site directed mutagenesis kit (NEB, catalog no. F541) as described earlier^14^. The primers used are listed in Supplementary Table 1. The pictorial representation of ERα domain structure and the plasmids encoding different domains is shown in Supplementary Fig. 4b,c. Different WDR13 isoforms are pictorially depicted in Supplementary Fig. 4d. Co-localization was performed in Ishikawa cells cultured in DMEM. GFP-ERα and FLAG-Wdr13S were co-transfected using Lipofectamine 2000 (Thermo Fisher, catalog no. 11668027, USA) as per manufacturer’s instructions. WDR13 was visualized by anti-FLAG primary antibody (Sigma, Catalogue no. F3165, USA) and Cy3 labelled secondary antibody. DAPI was used to visualize nucleus. Images were captured on confocal laser microscope (Leica microsystems).

### Reporter assay

Interaction of WDR13 with ERE promoter was assessed using, luciferase reporter system as described earlier^14^. Flag-Wdr13 and ERE luciferase reporter vector at 250 ng each were transfected by Lipofectamine 2000 Transfection Reagent (Thermo Fisher, catalog no. 11668027, USA). After 24 h of transfection, either E2 at 10 nM and/or Trichostatin A (Sigma, catalog no. T8552, USA) at 50 nM were added to the wells. After 18 h of treatment, cells were washed with PBS and then lysed in Promega reporter lysis buffer. Luminescence was measured by luminometer (LUMAC Biocounter M2000) after mixing cell lysate and luciferase substrate. The readings were normalized by cell protein content as measured by Thermo Fischer Micro BCA protein assay kit (Thermo Fischer, catalog no. 23235, USA).

### Estradiol, progesterone and insulin measurements

Blood was drawn from orbital sinus of the mice. Cayman Estradiol ELISA kit (Cayman Chemical, Catalog no. 582251, USA) was utilized to measure plasma estradiol levels. Insulin estimation was done using Rat/Mouse Insulin ELISA kit (Millipore, catalog no EZRMI-13K, USA). Plasma progesterone was estimated by using Cayman Progesterone ELISA Kit (catalog no. 582601, USA).

### DES treatment

Mice were treated with an estrogen analogue, diethylstilbestrol (DES) following an established and standard protocol^35^. Briefly, female *Wdr13*^+*/*+^ and *Wdr13*^−/−^ neonatal pups were treated with DES by subcutaneously injecting 2 μg per day for each pup from day 1 to day 5 after their birth (Fig. 7a). Oil-injected animals were considered as controls. Uteri were collected from treated and control animals at different required time intervals.

### Echo MRI analysis

Body weight analysis that includes information on fat mass and lean mass percentages was analysed at 3 months of age using EchoMRI-500 Body Composition Analyzer.

### Statistics

Unpaired Student’s *t* test was used with significance at p < 0.05. Data is represented as mean ± SEM. * indicates p-value less than 0.05, ** indicates p-value less than 0.01 and *** indicates p-value less than 0.001. For DES treated experiments, two-way Anova test was performed to find the statistical significance. The values indicated by asterisk (***) differ significantly at p < 0.001 and (*) differ significantly at p < 0.05.

## Supplementary information

Supplementary Figure 1.

Supplementary Figure 2.

Supplementary Figure 3.

Supplementary Figure 4.

Supplementary Figure 5.

Supplementary Figure 6.

Supplementary Figure 7.

Supplementary Table 1.

Supplementary Captions.
