# Supplementary figures and images for: Molecular characterization of Wdr13 knockout female mice uteri: a model for human endometrial hyperplasia
Source: Sci Rep. 2020 Sep 3;10:14621. doi: 10.1038/s41598-020-70773-w (PMC7471898; doi:10.1038/s41598-020-70773-w)

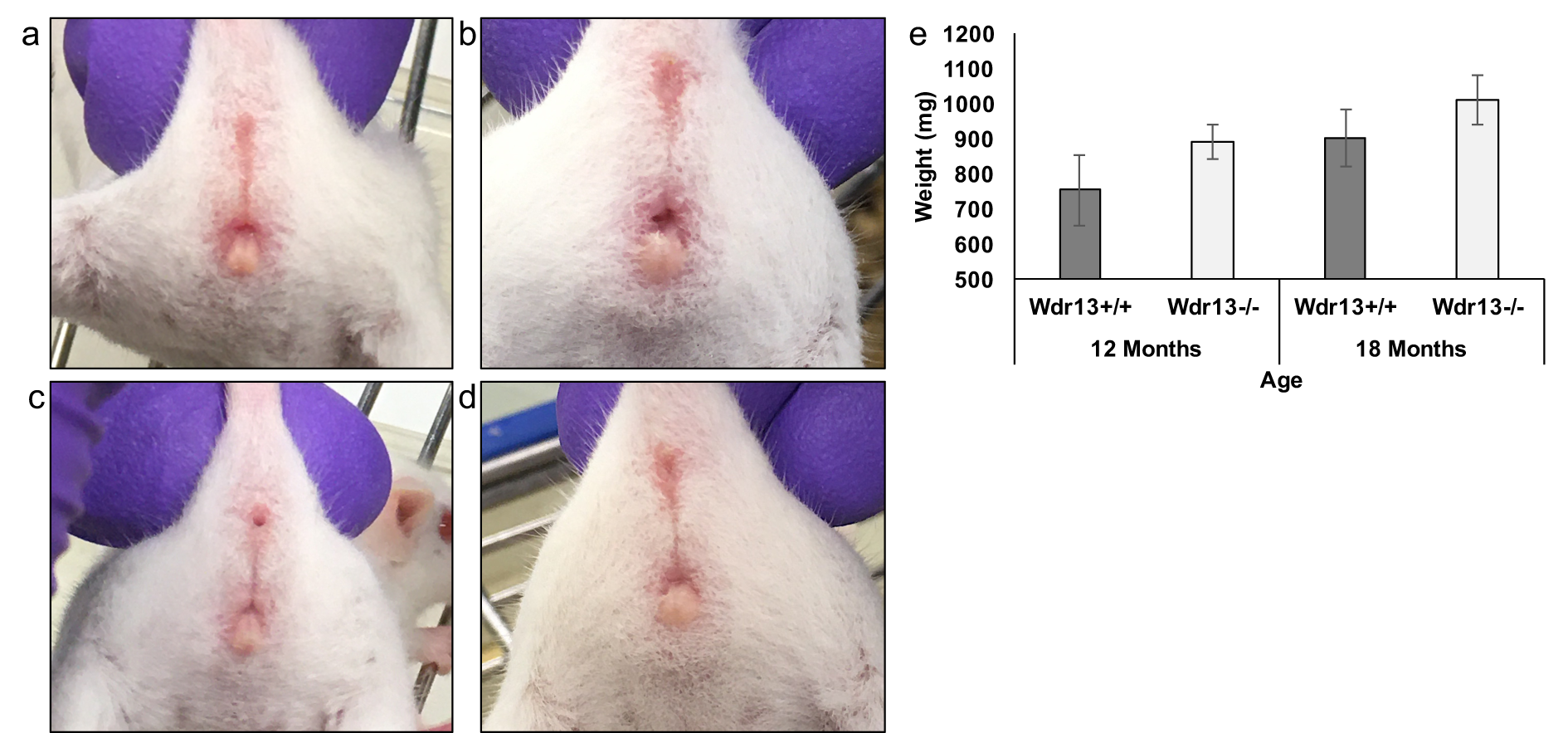

Supplement: Supplementary file 1 — Supplementary Figure 1. [file 41598_2020_70773_MOESM1_ESM.tif]

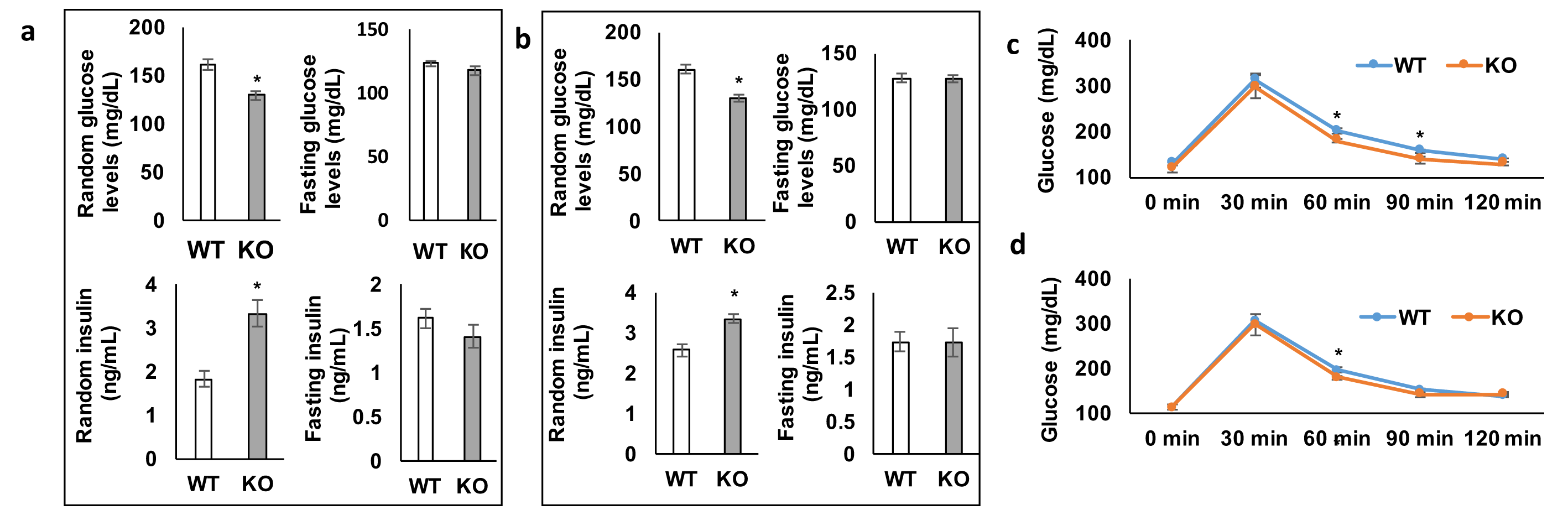

Supplement: Supplementary file 2 — Supplementary Figure 2. [file 41598_2020_70773_MOESM2_ESM.tif]

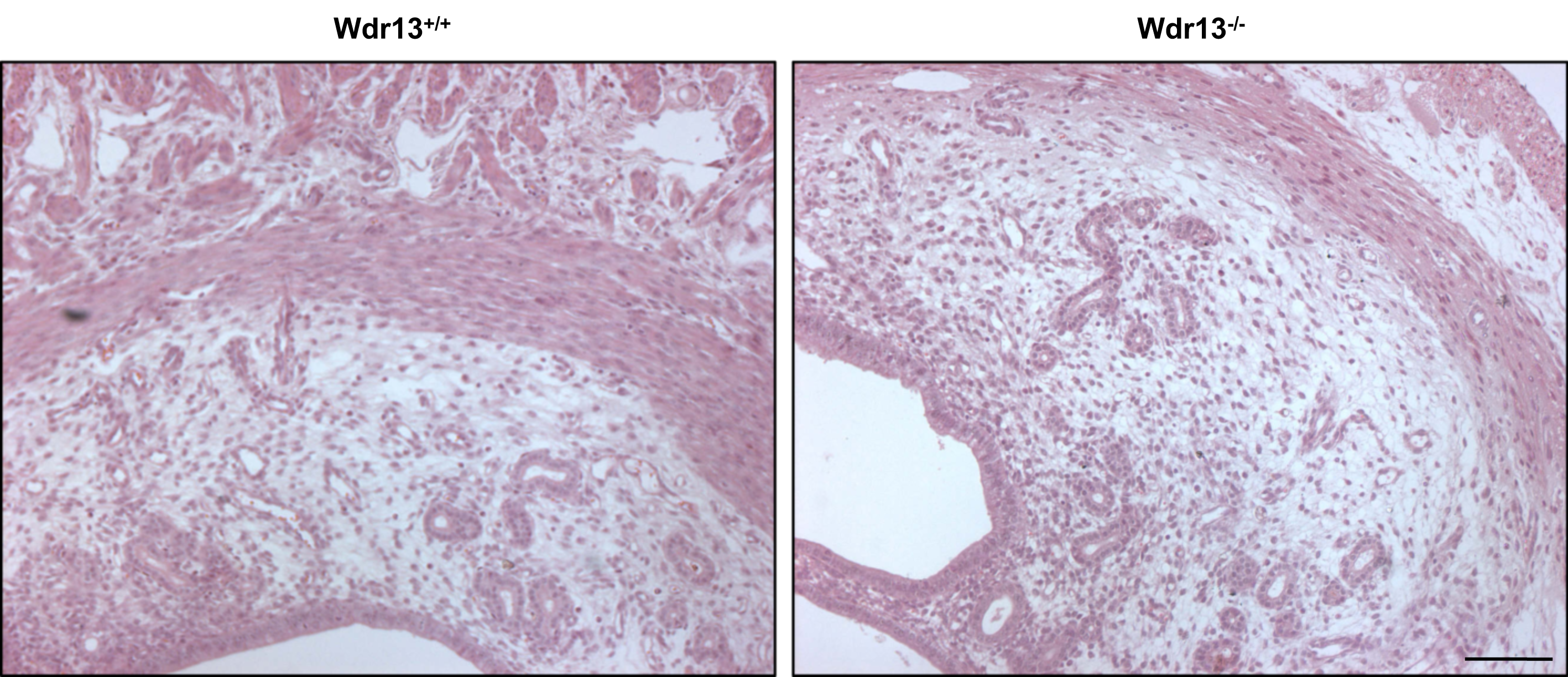

Supplement: Supplementary file 3 — Supplementary Figure 3. [file 41598_2020_70773_MOESM3_ESM.tif]

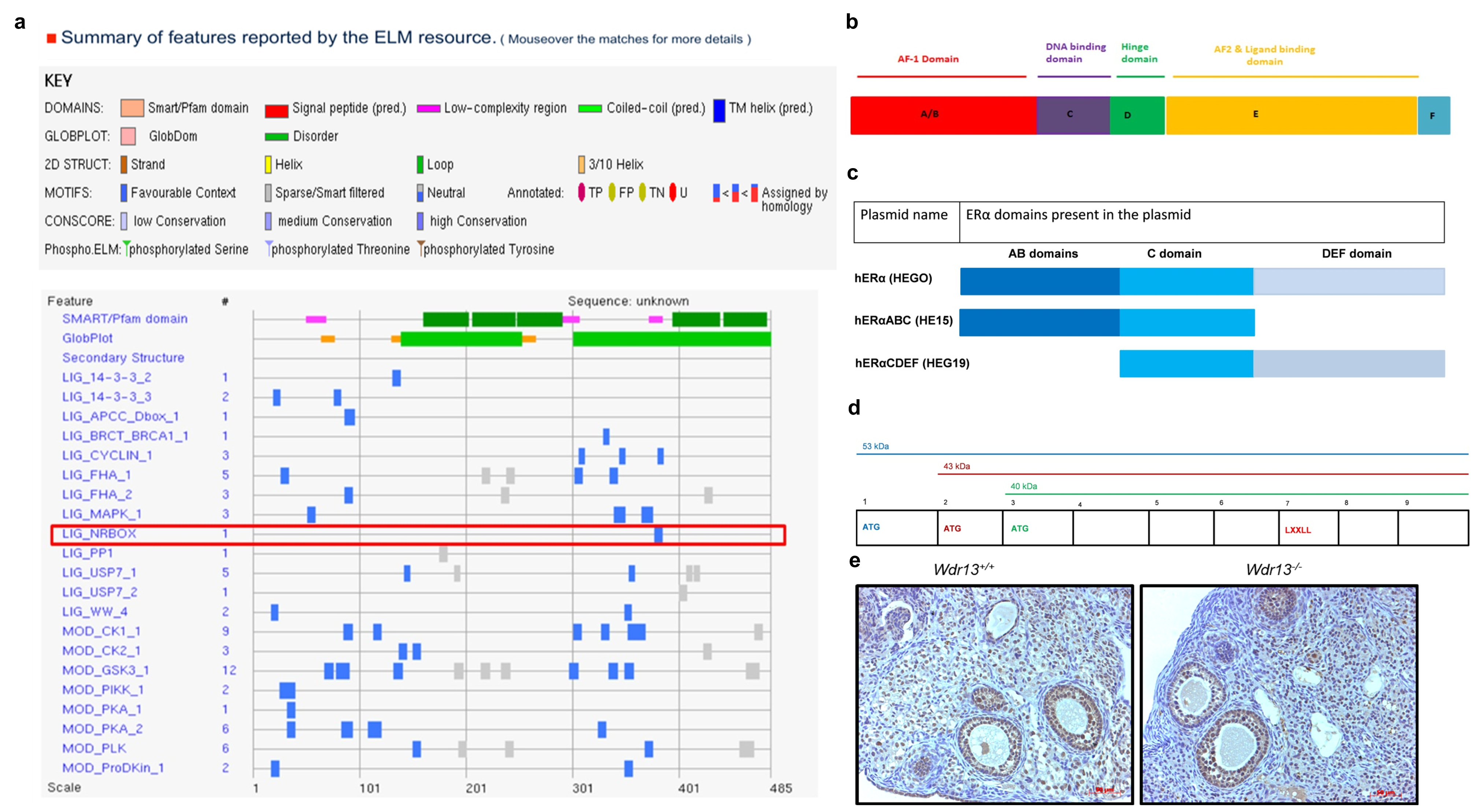

Supplement: Supplementary file 4 — Supplementary Figure 4. [file 41598_2020_70773_MOESM4_ESM.tif]

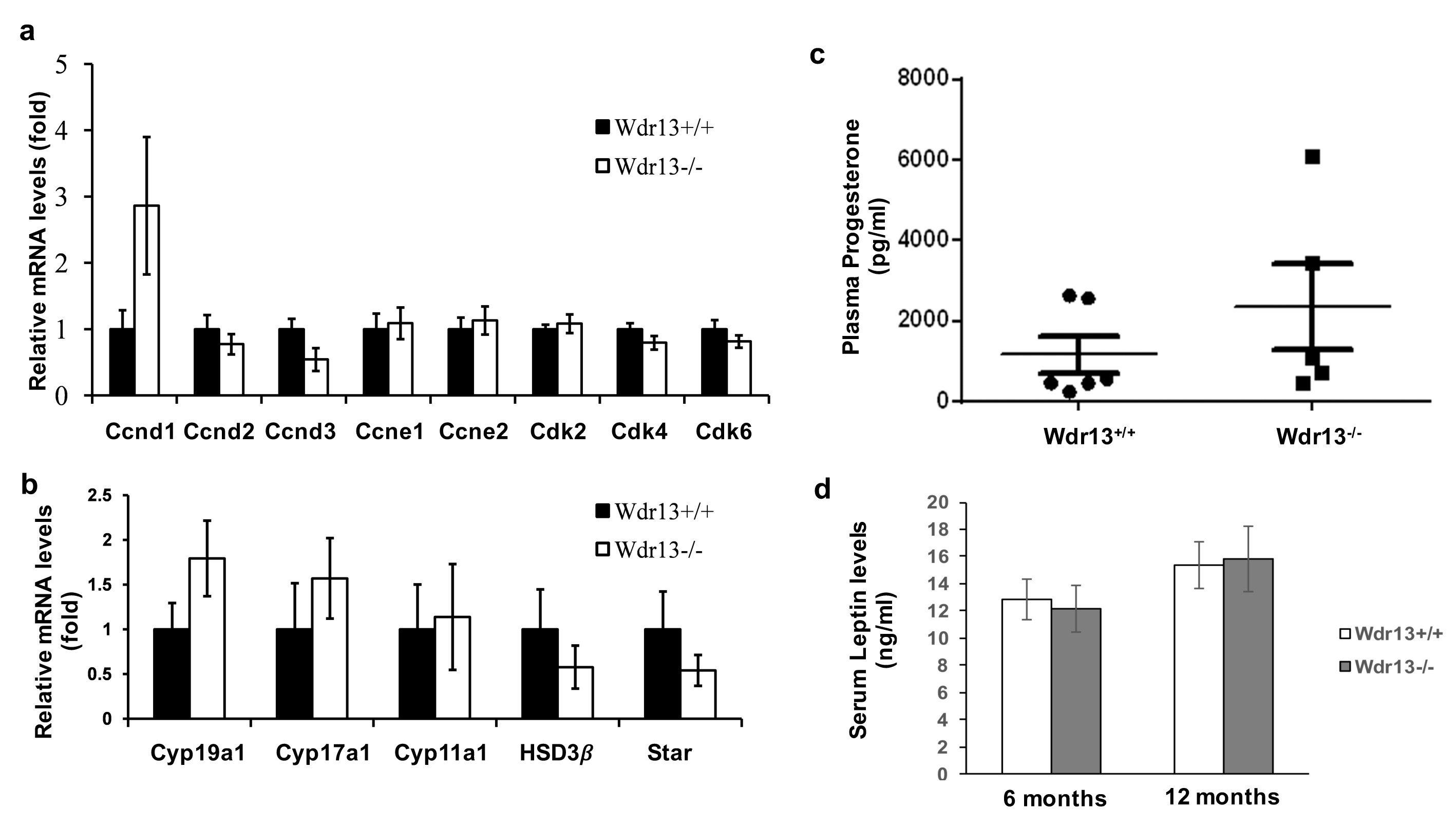

Supplement: Supplementary file 5 — Supplementary Figure 5. [file 41598_2020_70773_MOESM5_ESM.tif]

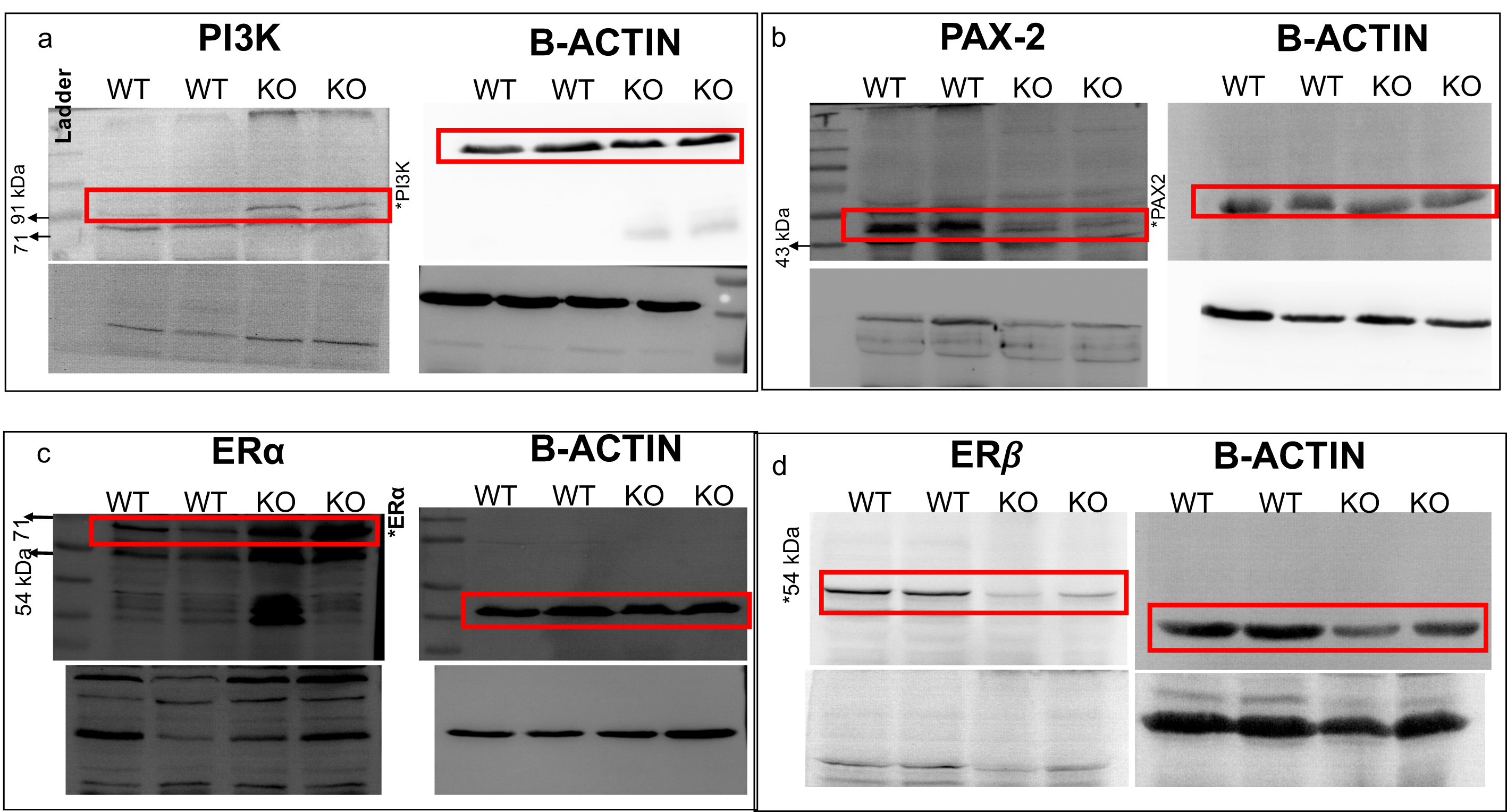

Supplement: Supplementary file 6 — Supplementary Figure 6. [file 41598_2020_70773_MOESM6_ESM.tif]

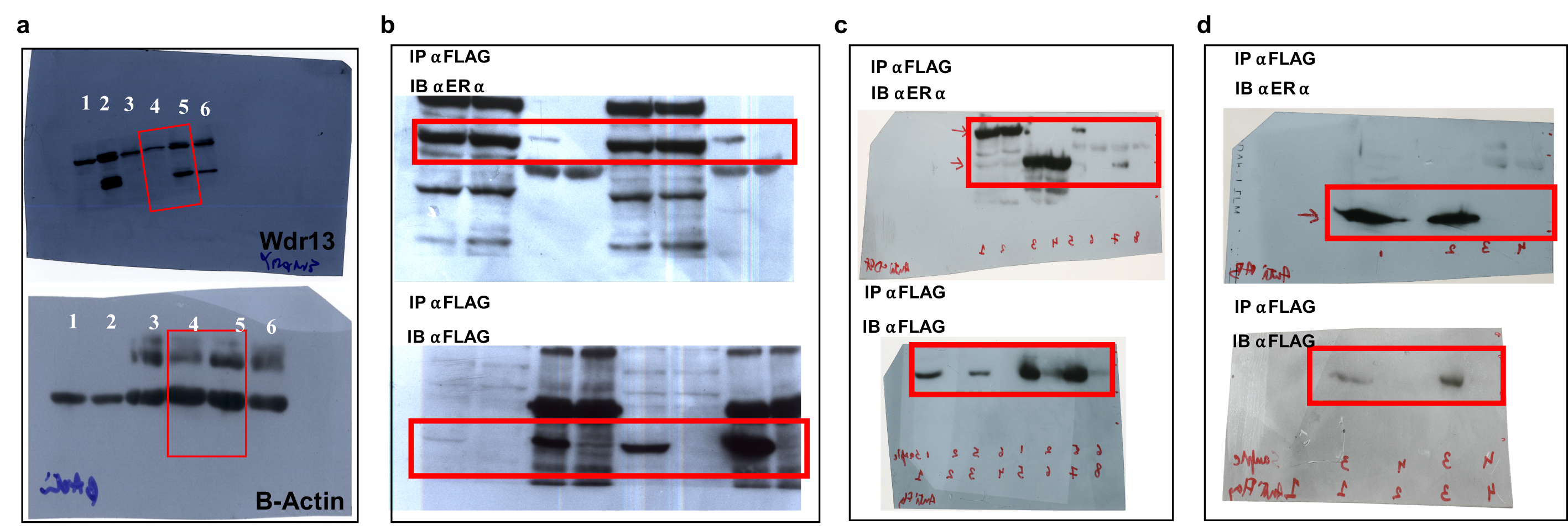

Supplement: Supplementary file 7 — Supplementary Figure 7. [file 41598_2020_70773_MOESM7_ESM.tif]
