# Supplementary material for: Molecular characterization of Wdr13 knockout female mice uteri: a model for human endometrial hyperplasia
Source: Sci Rep. 2020 Sep 3;10:14621. doi: 10.1038/s41598-020-70773-w (PMC7471898; doi:10.1038/s41598-020-70773-w)
Supplement: Supplementary file 8 — Supplementary Table 1. [file 41598_2020_70773_MOESM8_ESM.docx]

**Supplementary Table 1 List of primers**

| **S.No** | **Primer name** | **Primer use** | **Primer sequences (5’-3’)** |
| --- | --- | --- | --- |
| 1 | Rpl13aF (Mouse)  Rpl13aR  (house keeping gene) | qPCR | TTGCTTACCTGGGGCGTCT  CCTTTTCCTTCCGTTTCTCCTC |
| 2 | Cyclin D1F  Cyclin D1R | qPCR | CCCTTGGGGACATGTTGTTA  CCCTACTCTCAGGGTGATGC |
| 3 | Cyclin D2F  Cyclin D2R | qPCR | CTAGTGCATATGCCCCCTTT  CGTTTCTGATTCCTCCTTGG |
| 4 | Cyclin D3F  Cyclin D3R | qPCR | CCCTCCCTTTCTTGTCTTCC  TGAAAGCCCTTGGTCTGAGT |
| 5 | Cyclin E1F  Cyclin E1R | qPCR | CCATTGCCTCCAAAGACAGT  CACTTCCATCCAAGGCATCT |
| 6 | Cyclin E2F  Cyclin E2R | qPCR | GACCTAAACTATTTTCTTATGGCAGA  AGACAAGGTTCATGCCTGGT |
| 7 | Cdk2F  Cdk2R | qPCR | TGGTCTGTTCATCGTGGTTC  GAGGCCCTCTGACAACTCAA |
| 8 | Cdk4F  Cdk4R | qPCR | CAGCACTCCTACCTGCACAA  AGGAGAGGTGGGGACTTGTT |
| 9 | Cdk6F  Cdk6R | qPCR | CTCTTCAAAGCCCTGAGCTG  CCTGGAGTCACCACTCACCT |
| 10 | p21F  p21R | qPCR | GCCTTAGCCCTCACTCTGTG  AGCTGGCCTTAGAGGTGACA |
| 11 | Cyclin G2F  Cyclin G2R | qPCR | CCGGTCCGTGACGCC  AGTTCAACAATCCGAAAAGCTGA |
| 12 | RibF HUMAN  RibR HUMAN  (house keeping gene) | qPCR | GTGTTCGACAATGGCAGCAT  GACACCCTCCAGGAAGCGA |
| 13 | p21F HUMAN  p21R HUMAN | qPCR | CTGCCCAAGCTCTACCTTCC  TGGAGAAGATCAGCCGGCG |
| 14 | Wdr13F HUMAN  Wdr13R HUMAN | qPCR | GACTGGAACTGCGACTGACA  ATGCTTGGGGTGCAAGTAAC |
| 15 | Cyclin G2F HUMAN  Cyclin G2R HUMAN | qPCR | ATCGTTTCAAGGCGCACAG  CAACCCCCCTCAGGTATCG |
| 16 | SDMF  SDMR | Site directed mutagenesis | CTGCCTCAACAAGGCCGCCCTCTACAGAGTGGTGG  CCACCACTCTGTAGAGGGCGGCCTTGTTGAGGCAG |
| 17 | ERαF  ERαR | qPCR | CGCCTTCTACAGGTCTAAT  GGTTCTTGTCAATGGTGC |
