# Supplementary material for: Molecular characterization of Wdr13 knockout female mice uteri: a model for human endometrial hyperplasia
Source: Sci Rep. 2020 Sep 3;10:14621. doi: 10.1038/s41598-020-70773-w (PMC7471898; doi:10.1038/s41598-020-70773-w)
Supplement: Supplementary file 9 — Supplementary Captions. [file 41598_2020_70773_MOESM9_ESM.docx]

**Supplementary Figure 1 Estrus cycle and uterine weights of *Wdr13* knockout mice**.

Four stages of estrus cycle in *Wdr13* mutant mice. The four stages of estrus cycle are shown here as: - (a) proestrus, (b) estrus, (c) metestrus, (d) diestrus. (e) Uterine weights were measured for *Wdr13* wildtype and knockout mice at 12 and 18 month age old mice. No significant difference was found between the weights of wildtype and knockout mice

**Supplementary Figure 2 Random and fasting glucose and insulin levels in wildtype and mutant mice**

(a,b) Random and fasting glucose and insulin levels were measured in *Wdr13* wildtype and knockout mice at 12 and 18 months of age. A significant decrease and increase in the levels of random glucose and insulin were found in the mutant mice (n=6). (c,d) Glucose clearance test was performed for the wildtype and mutant mice at 12 and 18 months of age. Mutant mice showed better clearance of glucose compared to the wildtype mice. The values indicated by asterisk (*) differ significantly at p<0.05.

**Supplementary Figure 3 Histomorphological examination of uterine tissue at 3 months of age in wildtype and mutant mice.**

H&E staining was performed for the wildtype and knockout uterine tissue section at 3 months of age showed no abnormality in the uterine structure. Sections were observed at 100X magnification and the scale bar represents 100 μm.

**Supplementary Figure 4 Linear motif analysis, representation of ERα domains, WDR13 isoforms and ki67 immuno-histochemical staining of wildtype and mutant ovaries.**

(a) Eukaryotic linear motif search was performed using elm database (<http://elm.eu.org/)>. WDR13 showed nuclear receptor box motif as predicted by the database (b,c) Pictorial representation of ERα domains, whole ERα and independent ERα domain encoding plasmids (d) *Wdr13* cDNA with 9 exons encoding three isoforms. (e) Immuno-histochemical staining using ki-67 antibody revealed no abnormal morphology between wildtype and mutant mice ovaries.

**Supplementary Figure 5 Analysis of cell cycle regulators, important enzymes in estradiol synthesis, progesterone and leptin levels in wildtype and mutant mice**

(a) Gene expression (mRNA) levels of cell cycle regulators (cyclin D1, cyclin D2, cyclin D3, cyclin E1, cyclin E2, cyclin dependent kinase 2, cyclin dependent kinase 4 and cyclin dependent kinase 6) were analyzed in *Wdr13* knockout and wild type mice uteri (n=5). No significant difference was found in the levels between wildtype and mutant mice. (b) mRNA levels of enzymes (Cyp19a1, Star, Cyp17a1, Hsd3β, Cyp11a1) that mediate synthesis of estradiol, were analyzed using QPCR. No significant difference in the expression levels were found between mutant and wild type mice ovaries (n=5). (c,d) ELISA technique was employed to find the levels of progesterone and leptin. No significant difference was obtained in progesterone and leptin levels between mutant and wild type mice at 12 & 18 months of age and 6 & 12 months of age respectively (n=6).

**Supplementary Figure 6 Immunoblots for the proteins ERα, PI3K, PAX2 and ERβ in wildtype and mutant mice.**

(a-d) Full length immunoblots developed for analyzing proteins PI3K (91 kDa), PAX2 (43 kDa), ERα (71, 54 kDa) and ERβ (54 kDa) in wildtype and mutant mice were represented in the figure. Loading controls are β-actin (42 kDa). Red line box indicates corresponding image cropped to represent in the main figure. 3c,f.

**Supplementary Figure 7 Immunoblots of WDR13 expression in uterus and WDR13-ERα interaction studies.**

(a) Represents immunoblot corresponding to Fig.1a. Lane 1 to 6 correspond to WDR13 protein expression in *Wdr13* knockout brain tissue (lane 1), wildtype brain (lane 2), knockout uterus (lane 3 and 4), wildtype uterus (lane 5 and 6). (b) shows immunoblot corresponding to Fig. 5c where interaction of WDR13S with ERα was studied (independent of NR box motif) using anti-FLAG agarose beads to pull down ERα in both intact and mutated WDR13S overexpressing cells. (c) represents immunoblot corresponding to Fig. 5d to study WDR13 interaction with CDEF domain of ERα and whole ERα by pull down assay using anti-FLAG agarose beads. (d) indicates immunoblot corresponding to Fig. 5e showing no interaction between WDR13 and ABC domain of ERα. Red line box indicates image cropped to represent in the corresponding main figure as indicated in the legend.
